# Diploid mycelia of *Ustilago esculenta* fails to maintain sustainable proliferation in host plant

**DOI:** 10.3389/fmicb.2023.1199907

**Published:** 2023-07-24

**Authors:** Shiyu Li, Mengfei Yang, Tongfu Yao, Wenqiang Xia, Zihong Ye, Shangfa Zhang, Yipeng Li, Zhongjin Zhang, Ruiqi Song

**Affiliations:** ^1^Zhejiang Provincial Key Laboratory of Biometrology and Inspection & Quarantine, College of Life Sciences, China Jiliang University, Hangzhou, Zhejiang, China; ^2^Zhejiang Provincial Key Laboratory of Characteristic Aquatic Vegetable Breeding and Cultivation, Jinhua Academy of Agricultural Sciences, Jinhua, Zhejiang, China; ^3^Institute of Crop Science, College of Agriculture and Biotechnology, Zhejiang University, Hangzhou, Zhejiang, China

**Keywords:** *Ustilago esculenta*, plant tissue, teliospores, tissue expansion, karyogamy

## Abstract

Smut fungi display a uniform life cycle including two phases: a saprophytic phase *in vitro* and a parasitic phase in host plants. Several apathogenic smut fungi are found, lacking suitable hosts in their habitat. Interestingly, MT-type *Ustilago esculenta* was found to maintain a parasitic life, lacking the saprophytic phase. Its long period of asexual proliferation in plant tissue results in severe defects in certain functions. In this study, the growth dynamics of *U. esculenta* in plant tissues were carefully observed. The mycelia of T- and MT-type *U. esculenta* exhibit rapid growth after karyogamy and aggregate between cells. While T-type *U. esculenta* successfully forms teliospores after aggregation, the aggregated mycelia of MT-type *U. esculenta* gradually disappeared after a short period of massive proliferation. It may be resulted by the lack of nutrition such as glucose and sucrose. After overwintering, infected *Zizania latifolia* plants no longer contained diploid mycelia resulting from karyogamy. This indicated that diploid mycelia failed to survive in plant tissues. It seems that diploid mycelium only serves to generate teliospores. Notably, MT-type *U. esculenta* keeps the normal function of karyogamy, though it is not necessary for its asexual life in plant tissue. Further investigations are required to uncover the underlying mechanism, which would improve our understanding of the life cycle of smut fungi and help the breeding of *Z. latifolia*.

## 1. Introduction

*Ustilago esculenta* is a type of biotrophic fungus that obligately parasitizes the perennial herb, *Zizania latifolia*, inducing gall formation at the tip of the plant stem. There are two types of *U. esculenta*, namely teliospore (T) and mycelium & teliospore (MT). The infection of T-type *U. esculenta* induces the host plant to form smut gall with abundant teliospores (referred to as “gray jiaobai” in Chinese), while the infection of MT-type *U. esculenta* induces the generation of white and juicy gall without teliospores (referred to as “jiaobai” in Chinese; You et al., 2011; Zhang et al., 2012, 2014; Ning, 2013; Yan et al., 2013). Jiaobai were initially cultivated approximately 2,000 years ago in China and Japan as a traditional flavor. Additionally, in India, the wild smut gall growing in lakes and wetlands is called “kambong” and is consumed as an edible vegetable (Terrell and Batra, 1982; Kawagishi et al., 2006; Jose et al., 2016).

Like the life cycle of *Ustilago maydis*, T-type *U. esculenta* undergoes two stages, including the saprophytic yeast-like haploid stage followed by the parasitic dikaryon stage (Piepenbring et al., 2002; Zhang et al., 2017). During the parasitic stage, the mycelia grow in host plants and absorb nutrients via the biotrophic interface. The two cell nuclei of a dikaryon fuse at the later parasitic stage to form a diploid cell. Subsequently, diploid mycelia massively proliferate in plant tissues and aggregate in the apoplast cavities (Doehlemann et al., 2008; Tollot et al., 2016). These aggregating mycelia are highly entangled and embedded in gelatinous polysaccharide matrix (Snetselaar and Mims, 1994; Banuett and Herskowitz, 1996). Finally, the mycelia are ruptured to generate dark-colored teliospores (Feldbrügge et al., 2004). The germination of teliospores will produce haploids and start the saprophytic stage of smut fungi.

Mycelium & teliospore-type *U. esculenta* can proliferate in plant tissues, but cannot generate teliospores at the later stage of infection (You et al., 2011; Zhang et al., 2017). Teliospores and the haploid cells generated by it are important for the entire life cycle of *U. esculenta*. Teliospores enable survival in harsh environments, while haploid cells are responsible for penetrating the plant epidermis and infecting new host plants after mating. It is a common way for T-type *U. esculenta* and other smut fungi to expand their population. Without teliospores, MT-type *U. esculenta* can only overwinter in plant tissues and infect the offspring of host plants in the following year (Zhang et al., 2012; Jose et al., 2016). Consequently, the reproduction of MT-type *U. esculenta* heavily relies on host plants. The mutualistic relationship between MT-type *U. esculenta* and *Z. latifolia* is very special because MT-type *U. esculenta* does not contribute to the viability of *Z. latifolia.* In other words, *Z. latifolia* can easily live without MT-type *U. esculenta*, whereas MT-type *U. esculenta* is susceptible to extinction without the cultivation of jiaobai by human beings. The differentiation between T- and MT-type *U. esculenta* might happen several thousand years ago, when humans started to cultivate and consume *Z. latifolia* with MT-type *U. esculenta*. It provides an excellent example for studying the transformation from a parasite to a mutualistic symbiont.

Mycelium & teliospore-type *U. esculenta* has maintained a parasitic life in plant tissues for over 2,000 years, whereas T-type *U. esculenta* needs to constantly switch between saprophytic haploids and parasitic mycelia. However, it remains largely unknown how T- and MT-type *U. esculenta* reside in plant tissues. In our previous research, two single nucleotide polymorphism (SNP) sites were identified in the internal transcribed spacer (ITS) sequence, distinguishing T- and MT-type. Probes and primers were designed based on the SNP sites, and quantitative PCR (qPCR) was conducted to identify and quantify the presence of T- and MT-type *U. esculenta*. This technique enables us to detect changes in *U. esculenta* abundance in different plant tissue at various developmental stages. Moreover, on observing tissue sections, it was found that the massive proliferation of *U. esculenta* coincides with the mycelia aggregation. The Regulator of sporogenesis 1 (*Ros1*) gene, a member of the WOPR family that serves as important transcription regulators in fungi, is indispensable for teliospore formation. A *Ros1*-deficient strain remains in the dikaryon stage, unable to undergo karyogamy (Tollot et al., 2016). Analysis of *Ros1* expression and DAPI staining confirmed the occurrence of karyogamy before massive proliferation. The present investigation enhances our understanding of the effect of nutrients, as the decreased biomass of MT-type *U. esculenta* after karyogamy may be caused by insufficient carbon source.

## 2. Materials and methods

### 2.1. Experimental materials

*Zizania latifolia* plants infected with MT- or T-type *U. esculenta* were acquired from the Aquatic Vegetable Research Base of Jinhua Institute of Agricultural Science (Zhejiang Province). The infected plants were transplanted on July 20, 2021, and sampled every 7 days starting from the transplanting date. Twenty plants were collected each time, including seven plants for analyzing the *U. esculenta* content in different plant issues, four plants for paraffin embedding, sectioning, and microscopic observation, and eight plants for studying the gene expression of *U. esculenta*. *Zizania latifolia* was sampled 11 times in total. *Zizania latifolia* plant samples after overwintering were collected on February 20, 2022.

### 2.2. Extraction of DNA and detection of *Ustilago esculenta* content

Approximately, 0.1 g of plant tissue was collected, frozen in liquid nitrogen, and ground in a mortar. Total DNA was extracted from roots, stems, and stem tips following the user manual of the plant genomic DNA extraction kit [Tiangen Biotech (Beijing) Co., Ltd., DP 305]. The DNA content was measured using a Nanodrop 2000 spectrophotometer (Thermo Fisher Scientific) and diluted to approximately 100 ng/μL using sterile water. The diluted DNA samples were stored at −20°C until further use.

For the detection of *U. esculenta* in *Z. latifolia*, specific probes and primers (Table 1) were designed based on the SNP on the ITS sequences. The primers and probes were synthesized by Nanjing GenScript Biotech Corporation. The qPCR was performed using the following items: 10 μL PerfectStart II Probe qPCR SuperMix (2x; Beijing TransGen Biotech, AQ 711, AQ 401), 1.5 μL T fluorescent probe (10 μmol·L^−1^), 1.5 μL MT fluorescent probe (10 μmol·L^−1^), 1.5 μL forward primer ITS-F (10 μmol·L^−1^), 1.5 μL reverse primer ITS-R (10 μmol·L^−1^), 1 μL DNA template extracted from *Z. latifolia* tissues, and 3 μL sterile nuclease-free water. The amplification was carried out for 40 cycles under the following conditions: pre-denaturation at 95°C for 2 min, denaturation at 95°C for 15 s, annealing at 52°C for 15 s, and extension at 72°C for 30 s.

**Table 1:** Probe and primer sequences of the ITS gene.

Name	Sequence (5′ → 3′)
ITS-F	AGCTACCCAATTTCAACACG
ITS-R	TTTAGACGACCGCATTACCA
Fluorescent probe FAM	CAGCTAACCGATG
Fluorescent probe IC	ACAGCCAACCGATGC

### 2.3. Paraffin embedding

The collected plant tissues were washed with normal saline, and fixed in a Carnoy fixative (3:1 ethanol: acetic acid) for 48 h. After fixation, the samples were washed thrice with PBS, and dehydrated in a series of ethanol solutions (70, 80, 90, 95, and 100%). They were then transparentized using a balanced mixture of ethanol: xylene for 15 min, and then xylene for 15 min. The transparentized samples were successively immersed in a 1:1 mixture of paraffin: xylene for 15 min, and in paraffin twice for 60 min. The samples were placed into embedding boxes, and melted paraffin was added to generate embedding blocks.

### 2.4. Sectioning and Periodic Acid-Schiff staining

The paraffin embedding block was sectioned at a thickness of 5 μm using a Leica rotating microtome (Leica RM2125 RTS). Sections were then dewaxed as instructed in the user manual of the environmentally friendly dewaxing solution (Wuhan Servicebio Co., Ltd., G1128). The dewaxed sections were stained as per the PAS staining kit (Wuhan Servicebio Co., Ltd., G1008). Upon staining, high-sugar *U. esculenta* in plant tissues got a dark red color while plant tissues had a light red color, thus distinguishing plant tissues from *U. esculenta*.

### 2.5. Analysis of sugars in plant tissues

6 mL of 80% ethanol was added to 0.2 g of fresh plant tissue and incubated at 65°C for 20 min. The supernatant was collected, and the sediment was subjected to two additional extraction cycles following the same procedure. The collected supernatant was combined and concentrated. The soluble sugars were analyzed using an HPLC system comprising the Waters 600 separation module and the Waters 2414 RI detector (Waters Corp, Milford, MA, United States; Zhao et al., 2020). Sugar content was determined by the external standard method with 10 biological replicates per sample, and sugar concentration was measured in mg·g^−1^ of fresh weight.

### 2.6. Cell nucleus staining

The paraffin embedding block was transversely sectioned at a thickness of 3 μm using a Leica rotating microtome (Leica RM2125 RTS), and sections were dewaxed as instructed in the user manual of the environmentally friendly dewaxing solution (Wuhan Servicebio Co., Ltd., G1128). Sections were stained using 10 μg/mL 4′,6-diamidino-2-phenylindole (DAPI) for 10 min at room temperature, rinsed with PBS, and sealed using a water-soluble sealer.

### 2.7. Microscopic observation and analysis

Plant tissue sections were observed using a Leica Aperio VERSA 8 scanner. PAS-stained tissue sections were observed under bright field conditions; DAPI staining was excited with 405 nm light, and observed at 440–460 nm.

Image J software^1^ was used to measure the average cell size and cell count in each cross-section.

### 2.8. RNA extraction and inverse transcription

Approximately, 0.1 g of plant tissue was collected, frozen in liquid nitrogen, and ground using a pestle and mortar. The total RNA of *U. esculenta* tissues was extracted according to the user manual of the FastPure Plant Total RNA Isolation kit (Polysaccharides & Polyphenolics–rich; Nanjing Vazyme Biotech Co., Ltd., RC 401) and stored at −80°C.

The integrity of RNA was verified by 1% agarose gel electrophoresis. High-quality RNA samples were selected for cDNA synthesis using the HiScript II Q RT SuperMix for qPCR (+gDNA wiper; Nanjing Vazyme Biotech Co., Ltd., R223), following the manufacturer’s instructions.

### 2.9. Gene expression assay

*β-actin* was selected as the reference gene. Table 2 lists the primer sequences for the *Ros1* gene and the reference gene. The relative expression levels were analyzed using the 2^-△△CT^ (Livak and Schmittgen, 2001) method. The qPCR reaction system contained the following items: 0.5 μL forward primer F (10 μmol·L^−1^), 0.5 μL reverse primer R (10 μmol·L^−1^), 1 μL cDNA template (100 mg·L^−1^), 10 μL MonAmp™ SYBR® Green qPCR mix (Mona Biotechnology Co. Ltd., MQ00401), and 8 μL sterile water. The amplification was carried out for 40 cycles under the following conditions: pre-denaturation at 95°C for 30 s, denaturation at 95°C for 10 s, annealing at 55°C for 15 s, and extension at 72°C for 30 s.

**Table 2:** Primer sequences of the *Ros1* and the reference gene.

Name	Sequence (5′ → 3′)
Ros1-F	GATGCCCAATGCTATGGA
Ros1-R	CGGAATGATGGTGATGAG
β-actin-F	CAATGGTTCGGGAATGTGC
β-actin-R	GGGATACTTGAGCGTGAGGA

## 3. Results

### 3.1. Variation in *Ustilago esculenta* content at different developmental stages of *Zizania latifolia*

**Figure 1:**
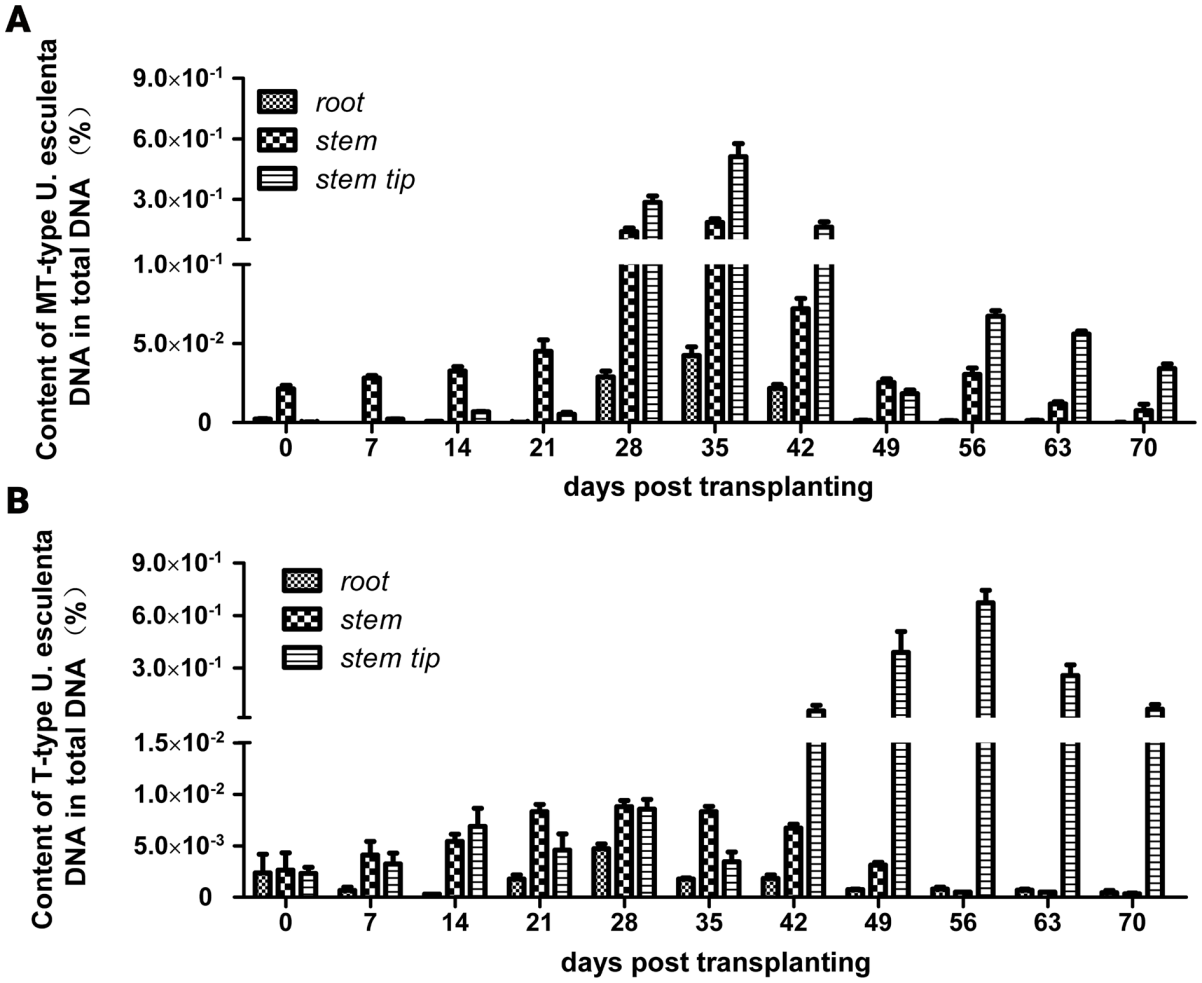
Variation in *Ustilago esculenta* content in plant tissues after transplanting. Roots, stems, and stem tip tissues of *Zizania latifolia* were collected, and their total DNA was extracted. The levels of T- and MT-type *U. esculenta* in plant tissues were, respectively, quantified using TaqMan qPCR. The DNA of *in vitro* cultured *U. esculenta* was extracted, diluted to different concentration levels, and used as standards for building a standard curve. The content of *U. esculenta* in the sample was calculated based on the standard curve; total DNA content was calculated using the 260 nm absorption band. The quotient between them was the content of *U. esculenta* in plant tissues. **(A)** The variation in MT-type *U. esculenta* content in white and juicy galls (eight biological replicates per experiment). **(B)** The variation in T-type *U. esculenta* content in smut galls (eight biological replicates per experiment).

Macroscopic phenotype observation revealed the following: (1) *Zizania latifolia* stems with MT-type *U. esculenta* significantly expanded on the 42nd day after transplanting, reaching a nearly edible size on the 120th day; (2) *Zizania latifolia* stems with T-type *U. esculenta* showed significant expansion on the 56th day after transplanting, and teliospores were observed on the 70th day. Smut galls induced by T-type *U. esculenta* were noticeably smaller compared to white and juicy galls induced by MT-type. The content of MT-type *U. esculenta* in plant stems was significantly higher than in roots and stem tips (value of *p* < 0.001) at the early stage (0–21 days after transplanting). Moreover, the content of MT-type *U. esculenta* in *Z. latifolia* stems peaked at least 0.1% on the 28 and 35th days after transplanting, whereas the content of T-type *U. esculenta* remained below 0.01%. *U. esculenta* exhibited extensive proliferation in stem tips and induced gall formation. The content profiles of T- and MT-type *U. esculenta* were similar in stem tips at the early stage. However, MT-type *U. esculenta* began to massively proliferate on the 28th day after transplanting, reaching peak content on the 35th day. Meanwhile, T-type massively proliferated since the 42nd day after transplanting, with peak content observed on the 56th day. The content of MT-type *U. esculenta* rapidly decreased after massive proliferation in stem tips, while T-type *U. esculenta* was kept at a high level. The gradual decline in the content of T-type *U. esculenta* at the later stage may be attributed to the low efficiency of DNA extraction from teliospores (Figure 1).

### 3.2. Microscopic observation of plant tissues at different stages

**Figure 2:**
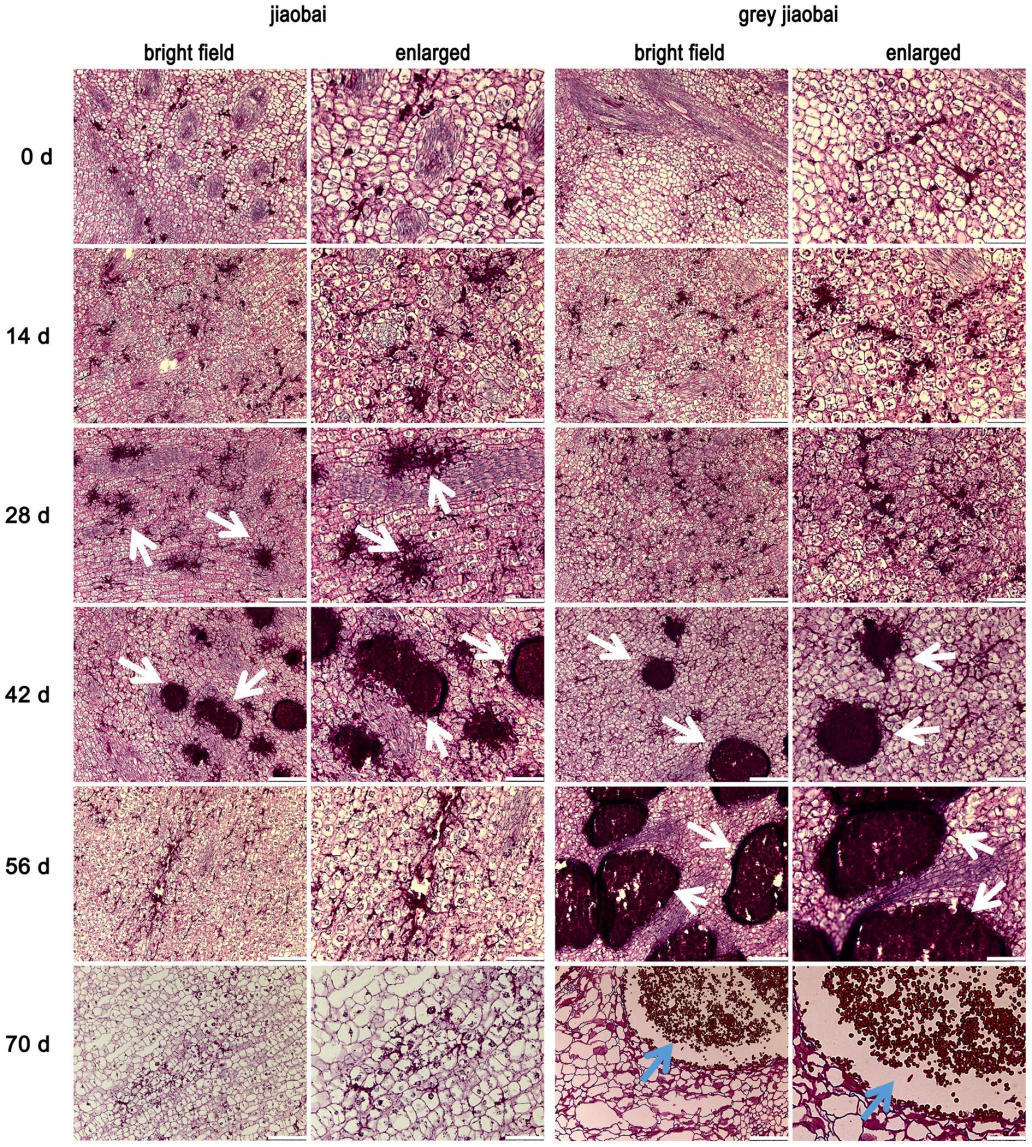
Stem tip tissue sections of *Zizania latifolia* at different stages after transplanting. Stem tip tissues of infected *Z. latifolia* were fixed, paraffin-embedded, and sectioned, followed by staining the plant tissues using Periodic Acid-Schiff (PAS). Microscopic examination was conducted under bright field conditions, and some areas were enlarged. *Ustilago esculenta* presented a dark red color, whereas plant tissues presented a light red color. Stem tip tissues were observed at different developmental stages (0, 14, 28, 42, 56, and 70 days after transplanting). *Ustilago esculenta* mycelia aggregated in plant tissues on the 28th and 42nd days after transplanting *Z. latifolia* infected with MT-type *U. esculenta*, and on the 42nd and 56th days after transplanting *Z. latifolia* infected with T-type *U. esculenta* (indicated by white arrows). Teliospores were massively generated in plant tissues 70 days after transplanting *Z. latifolia* infected with T-type *U. esculenta* (indicated by blue arrows). The scale bar represents 100 μm in the bright field image and 25 μm in the enlarged image.

To investigate the rapid decrease of MT-type *U. esculenta* on the 42nd day after transplanting, the stem tip of white and juicy galls and smut galls were sectioned and observed on 0, 14, 28, 42, 56, and 70 days after transplanting. *Ustilago esculenta* was PAS-stained into a dark red color (Figure 2). Initially, a small quantity of *U. esculenta* was scattered in plant tissues. A few mycelia of MT-type *U. esculenta* started to aggregate in *Z. latifolia* on the 28th day after transplanting; this phenomenon was observed a little later in T-type. The mycelia aggregation coincided with the rapid increase in *U. esculenta* content observed in section 3.1. The aggregated mycelia of MT-type *U. esculenta* gradually disappeared and diffused outward by the 56th day after transplanting. MT-type *U. esculenta* failed to form teliospores. In contrast, T-type *U. esculenta* formed a larger mycelia aggregation and generated teliospores by the 70th day after transplanting. These results indicate a critical difference between T- and MT-type *U. esculenta* that occurred after the mycelia aggregation.

### 3.3. Karyogamy analysis for *Ustilago esculenta*

**Figure 3:**
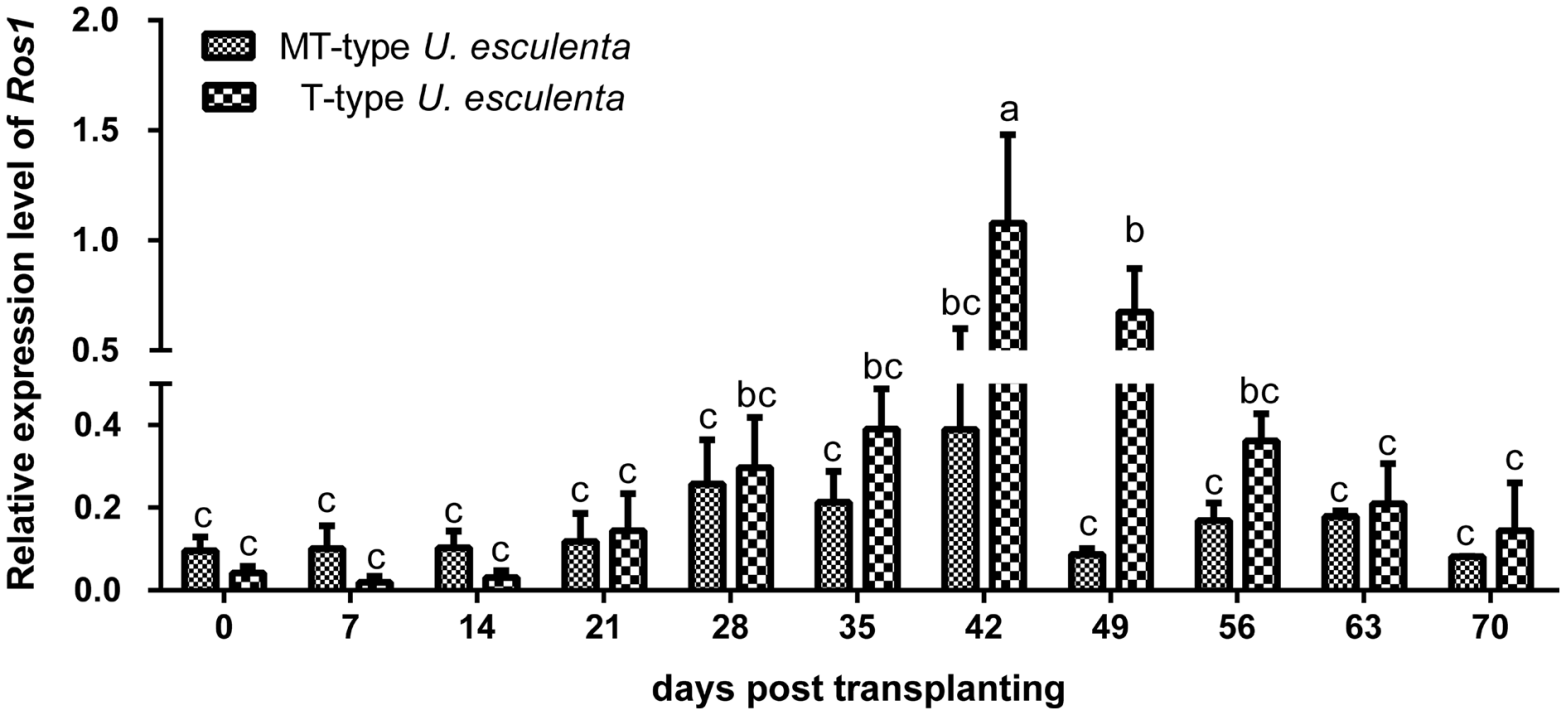
Variation in *Ros1* gene expression of *Ustilago esculenta* at different developmental stages Total RNA was extracted from the stem tip tissues of infected *Zizania latifolia* plants at different stages after transplanting. The expression of the *Ros1* gene was detected using RT-qPCR. The *β-actin* gene of *U. esculenta* was used as a reference gene, and eight biological replicates were performed per experiment. Different letters indicate significant differences (*p* < 0.05).

According to previous reports, massively proliferation and mycelia aggregation happened after karyogamy (Doehlemann et al., 2008; Tollot et al., 2016). The *Ros1* gene, belonging to the WOPR family, is closely associated with these processes (Tollot et al., 2016). In our study, the expression of the *Ros1* gene in T- and MT-type *U. esculenta* started to be upregulated on the 21st day after transplanting, and reached the maximum level on the 42nd day (Figure 3). The expression level in T-type *U. esculenta* was significantly higher on the 42nd and 49th days after transplanting compared to other time points, and it was significantly higher than that in MT-type. These results suggested that the karyogamy of *U. esculenta* might occur between the 21st and 42nd days after transplanting.

**Figure 4:**
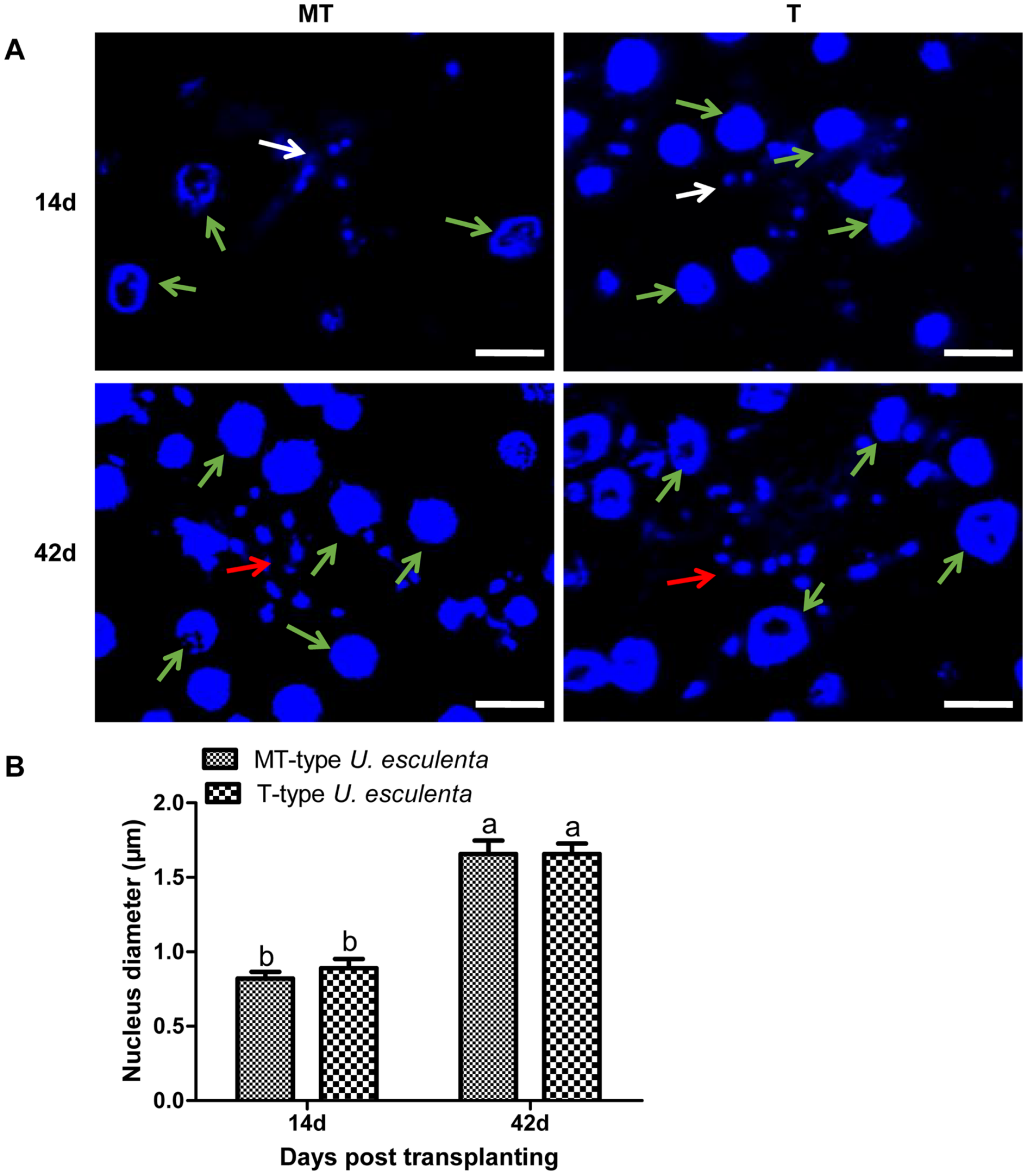
Observation of *Ustilago esculenta* cell nuclei at different developmental stages. Stem tip tissues of infected and uninfected *Z. latifolia* were selected on the 14th and 42nd days after transplanting and stained with DAPI to visualize the cell nucleus of MT- and T-type *U. esculenta*. **(A)** The larger cell nuclei (indicated by green arrows) belong to *Z. latifolia*, and the smaller cell nuclei belong to *U. esculenta*. On the 14th day after transplanting, a few pairs of small cell nuclei were observed (indicated by white arrows). On the 42nd day after transplanting, mycelia aggregated, and contiguous cell nuclei of *U. esculenta* were observed (indicated by red arrows). **(B)** A comparison of the cell nucleus diameter of *U. esculenta* at different stages was performed using Image J software. Eight plant tissue sections were measured at each time point, and at least 100 cell nuclei were observed in each tissue section. Different letters indicate significant differences (*p* < 0.05).

To further analyze the karyogamy of *U. esculenta*, stem tips of *Z. latifolia* on the 14th and 42nd days after transplanting were selected, and DAPI staining was performed to label cell nuclei (Figure 4). On the 14th day after transplanting, a few adjacent pairs of cell nuclei were observed in both MT- and T-type *U. esculenta*. Additionally, a few mononuclear cells were observed, possibly because the other cell nucleus was not in the current layer of sections. On the 42nd day after transplanting, the massive aggregation of mycelia made it difficult to distinguish the nuclei of different cells. Therefore, the size of cell nucleus was compared instead. The cell nucleus size ranged from 0.8 to 1.2 μm on the 14th day after transplanting, and significantly increased on the 42nd day (1.4–2.2 μm; *p* < 0.05). It suggested that karyogamy occurred in both MT- and T-type *U. esculenta*. The growth characteristics of *U. esculenta* undergo significant changes after karyogamy, including massively proliferation and mycelia aggregation.

### 3.4. Analysis of sugars in plant tissues

**Figure 5:**
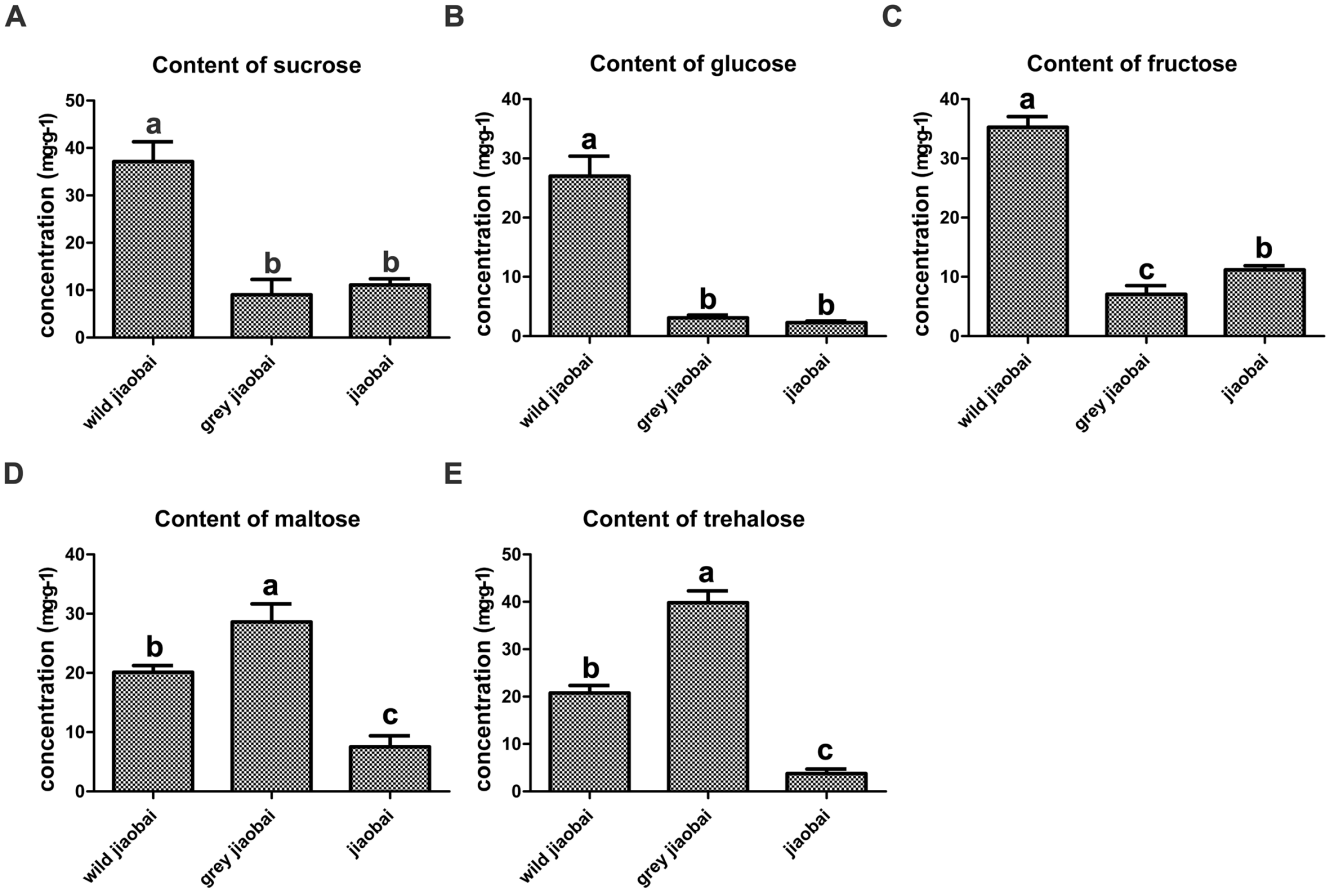
Analysis of sucrose **(A)**, glucose **(B)**, fructose **(C)**, maltose **(D)**, and trehalose **(E)** content in stems of gray jiaobai, jiaobai, and wild jiaobai 42 days after transplantation. Gray jiaobai refers to the smut gall produced by *Zizania latifolia* infected with T-type *Ustilago esculenta*; jiaobai refers to the white and juicy gall produced by *Z. latifolia* infected with T-type *U. esculenta*; wild jiaobai refers to the stem of uninfected *Z. latifolia*. Different letters indicate significant differences (*p* < 0.05).

The stems of infected and uninfected *Z. latifolia* plants were collected after 42 days of transplantation and analyzed. No matter what the type is (T or MT), the infected plants showed lower levels of fructose, glucose, maltose, and sucrose compared to the uninfected plants, indicating a high consumption rate of carbohydrates in the infected plant stems (Figure 5). On the other hand, the content of trehalose, which serves as an energy reserve of microbial carbohydrates, was significantly lower in MT-type *U. esculenta* than in T-type. Insufficient sugar supply in the stems appears to be the direct reason for the dramatic decrease in biomass of MT-type *U. esculenta* after 49 days of transplantation.

### 3.5. Observation of the cell nucleus of *Ustilago esculenta* after overwintering

**Figure 6:**
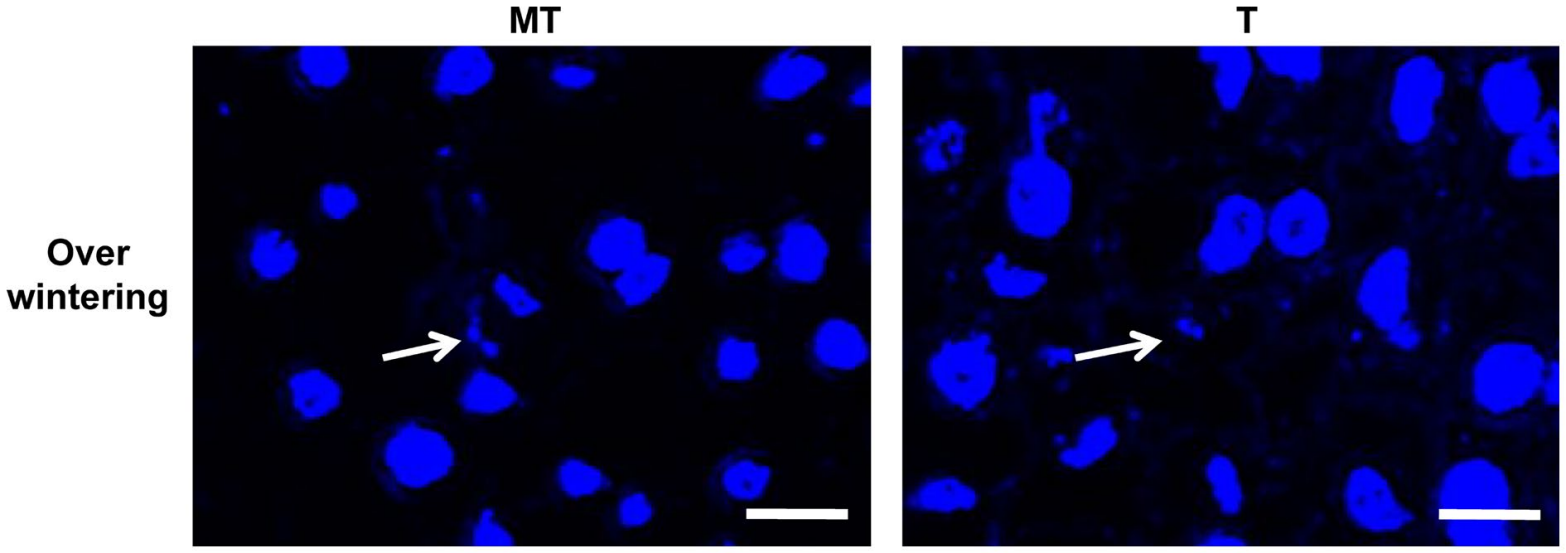
Observation of the cell nucleus of *Ustilago esculenta* after overwintering. Stem tip tissues of *Zizania latifolia* plants were collected on February 20, 2022, and stained with DAPI. A few pairs of small cell nuclei were observed (indicated by white arrows), but no cell nuclei with a diploid nucleus diameter were found.

As diploid mycelia will largely consume the nutrition in the host plant, the longevity of it in *Z. latifolia* plants was investigated. After overwintering, the plant tissues were collected, DAPI-stained, and observed to visualize the cell nuclei of *U. esculenta* (Figure 6). In both T- and MT-type *U. esculenta*, the dikaryotic form with a single nucleus diameter ranging from 0.7 to 1.2 μm was observed. This diameter was not significantly different from the cell nucleus diameter on the 14th day after transplanting in the previous year (*p* = 0.902). These findings indicate the absence of diploid mycelia generated in plant tissues in the previous year. Diploid mycelia of *U. esculenta* could not sustain long-term survival in *Z. latifolia* plants.

### 3.6. The effects of *Ustilago esculenta* on plant cells

**Figure 7:**
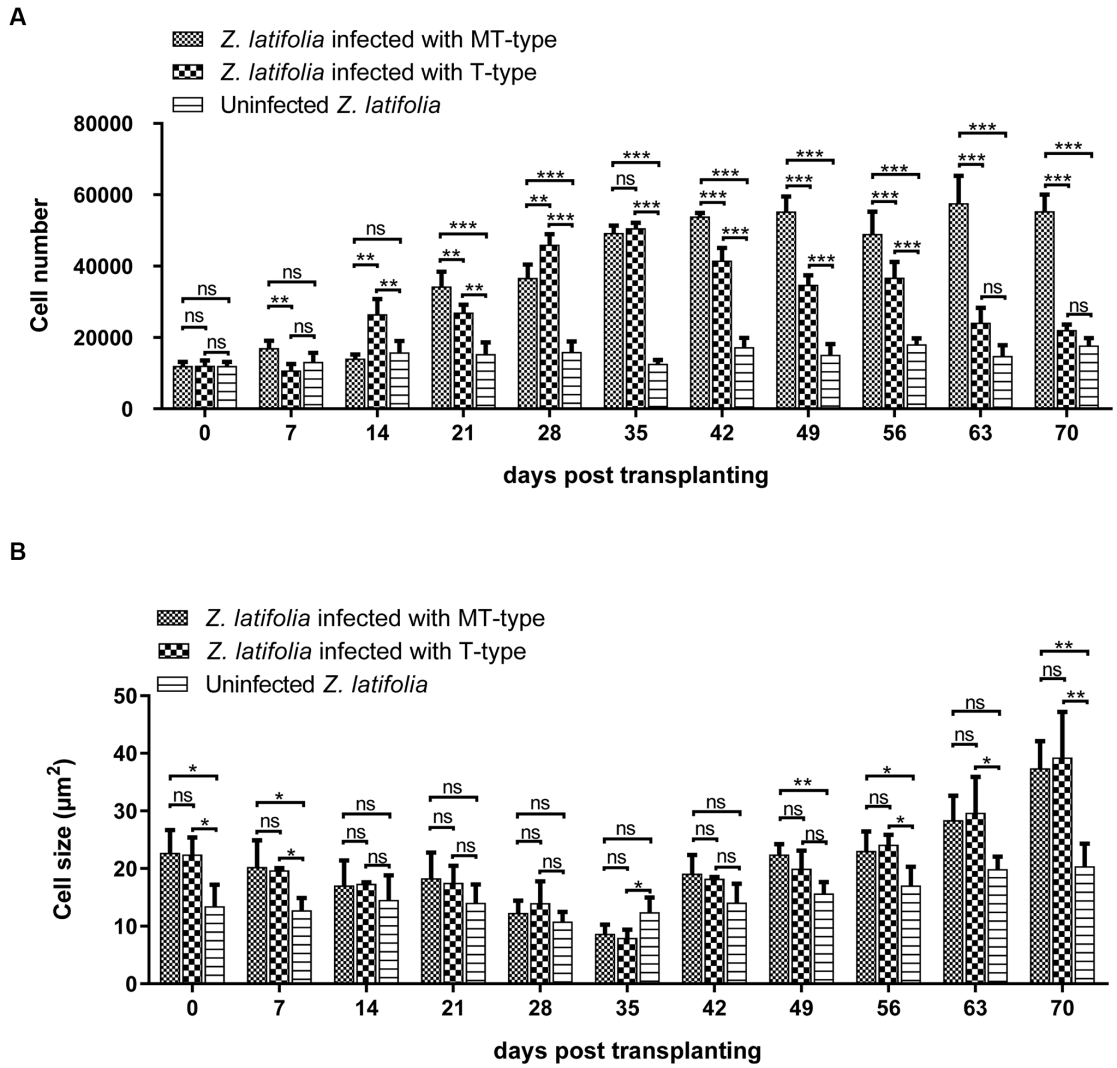
The effects of *Ustilago esculenta* growth on cell number and cell size of *Zizania latifolia* tissues. Stem tips were fixed, paraffin-embedded, and sectioned, followed by staining using the PAS. Microscopic examination was conducted under bright field conditions. The cell size **(A)** and cell number **(B)** were analyzed using Image J. The observed cross-sections represented the first segment of stem tip tissues, with the specialized aerenchyma located at the center. The aerenchyma was excluded from the analysis of the cell number and cell size. Eight biological replicates were conducted for each experiment [ns: no significant difference; ^*^p < 0.05; ^**^p < 0.01 ^***^p < 0.001 (student-*t* test)].

The growth characteristics of *U. esculenta* undergo significant changes after karyogamy, which can potentially impact host plants. To assess these effects, plant tissue sections were analyzed to measure cell number and cell size in the cross-section of the first segment of stem tip (Figure 7). *Zizania latifolia* infected with *U. esculenta* exhibited a similar cell number but larger cell size, resulting in an enlarged stem tip compared to uninfected *Z. latifolia* plants. Between day 0 and day 35 after transplanting, the number of cells in infected *Z. latifolia* gradually increased, although their stem tip size did not significantly increase. This was mainly due to two reasons: (1) a gradual decrease in average cell size; (2) the progressive occupation of aerenchyma by plant cells. Notably, the number of cells in *Z. latifolia* infected by MT-type *U. esculenta* remained nearly unchanged between the 42nd and 70th day after transplanting, whereas when infected with T-type, the number of cells gradually decreased due to the substantial occupation of space by mycelia aggregations and teliospores. Meanwhile, cell size significantly increased in infected *Z. latifolia* (both T- and MT-type) between the 42nd and 70th day after transplanting. The massive proliferation of T-type *U. esculenta* led to cell death at the later stage, while MT-type *U. esculenta* did not cause any cell death. However, the effects on other aspects of plant cells were similar between MT- and T-type *U. esculenta*.

## 4. Discussion

Jiaobai, a traditional delicacy cultivated in Asia, (Bunzel et al., 2002; Guo et al., 2007), relies on MT-type *U. esculenta* for the formation of white juicy galls. Understanding the life cycle of *U. esculenta* could help us to regulate the growth of *U. esculenta,* control the formation of galls, and increase its yield.

Mycelium & teliospore-type *U. esculenta* is significantly distinct from T-type *U. esculenta* owing to its exclusive reliance on asexual reproduction in plant tissues. Previous experiments have proven that MT-type *U. esculenta* could not generate teliospores (You et al., 2011; Zhang et al., 2014), subsequently leading to functional degeneration in aspects such as teliospore germination, haploid life, haploid fusion, and adaptability (Yang and Leu, 1978; Chan and Thrower, 1980; Zhang et al., 2017). In this study, a preliminary comparison was conducted to examine the growth characteristics of MT- and T-type *U. esculenta* in plant tissues.

This study showed that the content of MT-type *U. esculenta* gradually increased in plant tissues from day 0 to day 35 after transplanting, followed by a massive proliferation and mycelia aggregation after karyogamy. While the growth and development of T-type *U. esculenta* slightly lagged behind MT-type *U. esculenta*, they exhibited a similar overall pattern. However, at the later stage of plant growth, T-type *U. esculenta* continued to massively proliferate and generate teliospores, whereas the content of MT-type *U. esculenta* rapidly decreased, and the mycelia aggregation gradually disappeared in the stem of host plant. Combined with the expression of the *Ros1* gene and DAPI staining results, it was confirmed that the massive proliferation of *U. esculenta* and mycelia aggregation occurred soon after karyogamy, consistent with findings in *U. maydis* (Doehlemann et al., 2008; Tollot et al., 2016). This further supports the significant differences between dikaryotic mycelia and diploid mycelia in terms of living habits. To investigate the reasons behind this, analyses were performed on the content of important carbohydrates in the host plant’s stem at the later stage, leading to the conclusion that undernourishment may be the direct reason.

Our results showed that the massive proliferation of diploid mycelia after karyogamy heavily consumes nutrients in plant tissues, rendering them unable to maintain long-period survival. The formation of teliospores is a mechanism by which fungi survive across adverse conditions (Steinberg et al., 2017). Typically, when the fungus faces nutrient scarcity due to heavy consumption, it will initiate teliospore formation. However, in the case of *U. esculenta*, teliospore formation seems to be predetermined before the undernourishment. The massive proliferation of diploid mycelia before teliospore formation suggests that nutrients have not been exhausted at the time of karyogamy. Diploid mycelia undergo unlimited proliferation, depleting available nutrients. The destiny of teliospores formation is already determined upon the formation of diploid mycelium. In general, diploid mycelium serve as a specialized form for the formation of teliospores.

In the case of MT-type *U. esculenta*, because teliospores cannot be formed, and nutrients of the plant tissues are exhausted, the *U. esculenta* biomass eventually declines at the later stage. It is noteworthy that MT-type *U. esculenta* retains the normal functioning of karyogamy, though it is not necessary for its asexual reproduction in plant tissue. A few functions such as teliospore formation, germination, and the whole haploid life degenerate in MT-type *U. esculenta*. Recently, several studies have reported functional degeneration in the secretion of plant cell wall degrading enzymes and effectors, which are crucial for penetrating plant cuticles (Wang et al., 2022; Zhang et al., 2022). In contrast, MT-type maintains the functioning of karyogamy. One reasonable explanation is that the function of karyogamy has been selected during human cultivation. Karyogamy may be necessary for inducing white juicy galls consumed by human beings. This study revealed that plant tissue expansion occurs following the karyogamy of *U. esculenta*; however, *U. maydis* induces gall formation before karyogamy (Doehlemann et al., 2008). It has been reported that plant tissue becomes a strong “sink” during the expansion (Snetselaar and Mims, 1994; Banuett and Herskowitz, 1996), which may be related to the large nutrient consumption by mycelium after karyogamy. The swelling process of plant stem is influenced by both *U. esculenta* and *Z. latifolia* (Zhang et al., 2021; Li et al., 2022). Further studies are needed to explore these aspects in more detail.

At present, the exploration of factors contributing to the inability of *U. esculenta* to survive after karyogamy, particularly with regard to carbon source demand, has been limited in scope. To completely address this situation, follow-up verification is required. For example, transcriptome analysis can be performed before and after the karyogamy of MT- and T-type *U. esculenta*, to identify differentially expressed genes (León-Ramírez et al., 2017), and understand reasons why large quantities of *U. esculenta* cannot survive at the gene-expression level. Detection of the metabolome of stem tissues before and after karyogamy helps in predicting what kind of change in the nutritional environment of *U. esculenta* led to the decrease in biomass (Pang et al., 2021).

## Data availability statement

The original contributions presented in the study are included in the article/supplementary material, further inquiries can be directed to the corresponding author.

## Author contributions

ZY and SL: conceptualization and validation. SL, ZY, and ZZ: methodology. MY: software. SL and WX: formal analysis. SL and TY: resources and data curation. MY and TY: writing—original draft preparation. ZY, MY, WX, and SL: writing—review and editing. ZY: visualization, supervision, project administration, and funding acquisition. All authors contributed to the article and approved the submitted version.

## Funding

This work was supported by the National Natural Science Foundation of China (32202404 and 32100154), the Joint Funds of the National Natural Science Foundation of China (U20A2043), and Science and Technology Plan Project of Jinhua City (2020-2-010).

## Conflict of interest

The authors declare that the research was conducted in the absence of any commercial or financial relationships that could be construed as a potential conflict of interest.

## Publisher’s note

All claims expressed in this article are solely those of the authors and do not necessarily represent those of their affiliated organizations, or those of the publisher, the editors and the reviewers. Any product that may be evaluated in this article, or claim that may be made by its manufacturer, is not guaranteed or endorsed by the publisher.

## Footnotes

^1^ https://imagej.net/software/imagej/

## References

[ref1] Banuett F. Herskowitz I. (1996). Discrete developmental stages during teliospore formation in the corn smut fungus, *Ustilago maydis*. Development 122, 2965–2976. doi: 10.1242/dev.122.10.2965, PMID: 8898211

[ref2] Bunzel M. Allerdings E. Sinwell V. Ralph J. Steinhart H. (2002). Cell wall hydroxycinnamates in wild rice (*Zizania aquatica* L.) insoluble dietary fibre. Eur. Food Res. Tech. 214, 482–488. doi: 10.1007/s00217-002-0512-3

[ref3] Chan Y. S. Thrower L. B. (1980). The host-parasite relationship between *Zizania caduciflora* Turcz. And Ustilago esculenta p. Henn. Ii. Ustilago esculenta in culture. New Phytol. 85, 209–216. doi: 10.1111/j.1469-8137.1980.tb04462.x

[ref4] Doehlemann G. Wahl R. Vraneš M. de Vries R. P. Kämper J. Kahmann R. (2008). Establishment of compatibility in the *Ustilago maydis* maize pathosystem. J. Plant Physiol. 165, 29–40. doi: 10.1016/j.jplph.2007.05.016, PMID: 17905472

[ref5] Feldbrügge M. Kämper J. Steinberg G. Kahmann R. (2004). Regulation of mating and pathogenic development in *Ustilago maydis*. Curr. Opin. Microbiol. 7, 666–672. doi: 10.1016/j.mib.2004.10.006 15556041

[ref6] Guo H. B. Li S. M. Peng J. Ke W. D. (2007). *Zizania latifolia* Turcz. Cultivated in China. Genet. Resour. Crop. Evol. 54, 1211–1217. doi: 10.1007/s10722-006-9102-8

[ref7] Jose R. C. Goyari S. Louis B. Waikho S. D. Handique P. J. Talukdar N. C. (2016). Investigation on the biotrophic interaction of Ustilago esculenta on *Zizania latifolia* found in the indo-Burma biodiversity hotspot. Microb. Pathog. 98, 6–15. doi: 10.1016/j.micpath.2016.06.021, PMID: 27334294

[ref8] Kawagishi H. Hota K. Masuda K. Yamaguchi K. Yazawa K. Shibata K.. (2006). Osteoclast-forming suppressive compounds from Makomotake, *Zizania latifolia* infected with Ustilago esculenta. Biosci. Biotechnol. Biochem. 70, 2800–2802. doi: 10.1271/bbb.60376, PMID: 17090930

[ref9] León-Ramírez C. G. Cabrera-Ponce J. L. Martínez-Soto D. Sánchez-Arreguin A. Aréchiga-Carvajal E. T. Ruiz-Herrera J. (2017). Transcriptomic analysis of basidiocarp development in *Ustilago maydis* (DC) Cda. Fungal Genet. Biol. 101, 34–45. doi: 10.1016/j.fgb.2017.02.007, PMID: 28285895

[ref10] Li F. Zhang J. Zhong H. Chen J. (2022). Germicide fenaminosulf promots gall formation of *Zizania latifolia* without directly affecting the growth of endophytic fungus Ustilago esculenta. BMC Plant Biol. 22:418. doi: 10.1186/s12870-022-03803-6, PMID: 36042398 PMC9426258

[ref11] Livak K. J. Schmittgen T. D. (2001). Analysis of relative gene expression data using real-time quantitative PCR and the 2(-Delta Delta C(T)) method. Methods 25, 402–408. doi: 10.1006/meth.2001.1262 11846609

[ref12] Ning Y. (2013). The growth and heritability of Zizinia latifolia plants infected with sporidial strain of Ustilago esculenta. Physiol. Mol. Plant Pathol. 83, 75–83. doi: 10.1016/j.pmpp.2013.05.005

[ref13] Pang Z. Chen J. Wang T. Gao C. Li Z. Guo L.. (2021). Linking plant secondary metabolites and plant microbiomes: a review. Front. Plant Sci. 12:621276. doi: 10.3389/fpls.2021.621276, PMID: 33737943 PMC7961088

[ref14] Piepenbring M. Stoll M. Oberwinkler S. F. (2002). The generic position of *Ustilago maydis*, Ustilago scitaminea, and Ustilago esculenta (Ustilaginales). Mycol. Prog. 1, 71–80. doi: 10.1007/s11557-006-0006-y

[ref15] Snetselaar K. M. Mims C. W. (1994). Light and electron microscopy of *Ustilago maydis* hyphae in maize. Mycol. Res. 98, 347–355. doi: 10.1016/S0953-7562(09)80463-2

[ref16] Steinberg G. Peñalva M. A. Riquelme M. Wösten H. A. Harris S. D. (2017). Cell biology of hyphal growth. Microbiol. Spectr. 5, 1–34. doi: 10.1128/microbiolspec.FUNK-0034-2016 PMC11687463 28429675

[ref17] Terrell E. E. Batra L. R. (1982). Zizania latifolia and Ustilago esculenta, a grass-fungus association. Econ. Bot. 36, 274–285. doi: 10.1007/BF02858549

[ref18] Tollot M. Assmann D. Becker C. Altmüller J. Dutheil J. Y. Wegner C. E.. (2016). The WOPR protein Ros1 is a master regulator of sporogenesis and late effector gene expression in the maize pathogen *Ustilago maydis*. PLoS Pathog. 12:e1005697. doi: 10.1371/journal.ppat.1005697, PMID: 27332891 PMC4917244

[ref19] Wang S. Gao L. Yin Y. Zhang Y. Tang J. Cui H.. (2022). Transcriptome comparison between two strains of Ustilago esculenta during the mating. J. Fungi 9:32. doi: 10.3390/jof9010032, PMID: 36675853 PMC9862937

[ref20] Yan N. Wang X. Q. Xu X. F. Guo D. P. Wang Z. D. Zhang J. Z.. (2013). Plant growth and photosynthetic performance of *Zizania latifolia* are altered by endophytic Ustilago esculenta infection. Physiol. Mol. Plant Pathol. 83, 75–83. doi: 10.1016/j.pmpp.2013.05.005

[ref21] Yang H. C. Leu L. S. (1978). Formation and histopathology of galls induced by Ustilage esculenta in *Zizania latifolia*. Phytopathology 68, 1572–1576. doi: 10.1094/Phyto-68-1572

[ref22] You W. Liu Q. Zou K. Yu X. Cui H. Ye Z. (2011). Morphological and molecular differences in two strains of Ustilago esculenta. Curr. Microbiol. 62, 44–54. doi: 10.1007/s00284-010-9673-7, PMID: 20495805

[ref23] Zhang Z. Bian J. Zhang Y. Xia W. Li S. Ye Z. (2022). An endoglucanase secreted by Ustilago esculenta promotes fungal proliferation. J. Fungi 8:1050. doi: 10.3390/jof8101050, PMID: 36294616 PMC9605326

[ref24] Zhang Y. Cao Q. Hu P. Cui H. Yu X. Ye Z. (2017). Investigation on the differentiation of two Ustilago esculenta strains—implications of a relationship with the host phenotypes appearing in the fields. BMC Microbiol. 17:228. doi: 10.1186/s12866-017-1138-8, PMID: 29212471 PMC5719756

[ref25] Zhang J. Z. Chu F. Q. Guo D. P. Hyde K. D. Xie G. L. (2012). Cytology and ultrastructure of interactions between Ustilago esculenta and *Zizania latifolia*. Mycol. Prog. 11, 499–508. doi: 10.1007/s11557-011-0765-y

[ref26] Zhang J. Z. Chu F. Q. Guo D. P. Ojaghian M. R. Hyde K. D. (2014). The vacuoles containing multivesicular bodies: a new observation in interaction between Ustilago esculenta and *Zizania latifolia*. Eur. J. Plant Pathol. 138, 79–91. doi: 10.1007/s10658-013-0303-7

[ref27] Zhang Z. P. Song S. X. Liu Y. C. Zhu X. R. Jiang Y. F. Shi L. T.. (2021). Mixed transcriptome analysis revealed the possible interaction mechanisms between Zizania latifolia and *Ustilago esculenta* inducing jiaobai stem-gall formation. Int. J. Mol. Sci. 22:12258. doi: 10.3390/ijms222212258, PMID: 34830140 PMC8618054

[ref28] Zhao Y. Yue Z. Zhong X. Lei J. Tao P. Li B. (2020). Distribution of primary and secondary metabolites among the leaf layers of headed cabbage (*Brassica oleracea var. capitata*). Food Chem. 312:126028. doi: 10.1016/j.foodchem.2019.126028, PMID: 31896454

